# Supplementary material for: Transverse orbital angular momentum of amplitude perturbed fields
Source: Nanophotonics. 2025 Mar 19;14(6):777–84. doi: 10.1515/nanoph-2024-0595 (PMC11964137; doi:10.1515/nanoph-2024-0595)
Supplement: Supplementary file 1 — Supplementary Material Details [file j_nanoph-2024-0595_suppl_001.pdf]

Transverse orbital angular momentum of amplitude perturbed fields: supplement

S. W. Hancock†, N. Tripathi†, M. S. Le, A. Goffin, and H. M. Milchberg*

Institute for Research in Electronics and Applied Physics, University of Maryland, College Park, Maryland 20742, USA

† Co-first authors contributed equally to this work

* Corresponding author: milch@umd.edu

Fig. S1 below shows the experimental results for an $l = 1$ STOV with a $50\ \mu\mathrm{m}$ blockage at the range of data points plotted in Fig. 3(e) of the main text. The top row shows the measured intensity envelopes, $|A(x,\xi)|^2$, and their accompanying spatiotemporal phases, $\Phi(x,\xi)$ in the second row. The third row shows the calculated tOAM density, $M_y(x,\xi)$ obtained by the application of Eq. (1b) and Eq. (4).

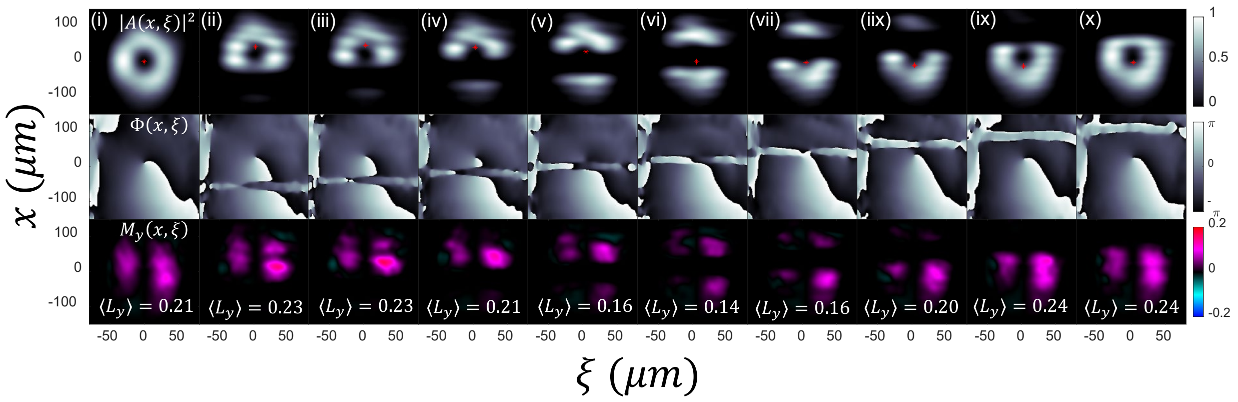

Fig. S1: Experimental results and simulations for 50 μm diameter tungsten wire perturbation of an $l = 1$ STOV pulse: rows, top to bottom, plot $|A(x,\xi)|^2$, $\Phi(x,\xi)$, and $M_y(x,\xi)$. Column (i) is unperturbed STOV, and columns (ii)-(v) show results for the wire centred at $x_0 = -80, -60, -40, -20, 0, 20, 40, 60, 80$ μm. The red dots mark the centres of energy and the measured tOAM is shown on the panels.
